# A129 CYP2C19 PHARMACOGENETIC TESTING IN PAEDIATRIC PATIENTS WITH EOSINOPHILIC ESOPHAGITIS INFLUENCES DOSING OF PROTON-PUMP-INHIBITORS AND RESPONSE TO THERAPY

**DOI:** 10.1093/jcag/gwab049.128

**Published:** 2022-02-21

**Authors:** K A Bortolin, I Cohn, S Da Silva, S Ito, P Marcon, N Afzal, S Scodellaro, R Verstegen, J Hulst

**Affiliations:** 1 The Hospital for Sick Children, Division of Gastroenterology, Hepatology and Nutrition, Toronto, ON, Canada; 2 The Hospital for Sick Children, Division of Clinical Pharmacology and Toxicology, Toronto, ON, Canada; 3 University of Toronto, Toronto, ON, Canada

## Abstract

**Background:**

Eosinophilic esophagitis (EoE) is a chronic inflammatory disorder that can be treated with a proton pump inhibitor (PPI). Pharmacogenetics (PGx) is the study of how variations in an individual’s genome influences drug response. Genetic variation in the metabolism gene CYP2C19 can produce differences in enzyme activity which is known to be a contributing factor for therapeutic failure with PPI treatment. Use of 2^nd^ generation PPI (rabeprazole) can be beneficial in some as this PPI is less effected by CYP2C19 metabolism. PGx has been studied in PPI therapy for peptic ulcer disease but has not been demonstrated in patients with EoE.

**Aims:**

To describe the CYP2C19 metabolism in patients with EoE on PPI and to estimate the clinical utility of PGx testing in directing subsequent changes in therapy with improvement in remission rates.

**Methods:**

Interim analyses of a single centre, non-interventional, ongoing descriptive pilot study investigating CYP2C19 metabolism in patients with EoE, as part of a larger PGx pilot study and EoE- AHEAD Registry Study at SickKids. Patients with EoE that were newly diagnosed and started PPI or those not in remission on current non-PPI therapy or not in remission on dose PPI (2 mg/kg/day, max 30 mg lansoprazole BID) were included. Active disease was defined as a peak eosinophil count >15/hpf.

**Results:**

37 patients met the inclusion criteria with completed PGx test; mean age was 13 years, 29(78%) were male, and 13(35%) had concurrent atopic disease. PGx testing showed that 12(32%) and 4(11%) were rapid (RM) and ultrarapid metabolizers (URM) respectively (**Fig.1**), which is significantly higher than the population average. Of this subgroup, 9 started rabeprazole, 3 had a lansoprazole dose increase, and 4 had no changes. Overall, changes in therapy based on PGx testing were made in 29(78%) patients, 8 are awaiting follow-up (**Fig 2**). Currently, the patients with available repeat biopsy results after PGx test-guided therapy changes is limited due COVID-19 related delays in endoscopies.

**Conclusions:**

The preliminary findings of our study using PGx to guide PPI dosing in pediatric patients with EoE demonstrate that PGx test results lead to a change in clinical management in most patients. In RM and URM, PGx results trigger an adjustment of PPI dose or type could lead to earlier disease remission in PPI-responsive patients, thereby optimizing PPI efficacy. PGx may support dose reduction in poor metabolizers aiming to avoid long-term adverse events.

Further correlation with endoscopy and histology findings of patients after PGx-guided therapy changes will follow. Furthermore, it is important to examine if CYP2C19 variant information available before PPI therapy further streamlines an initial phase of the treatment.

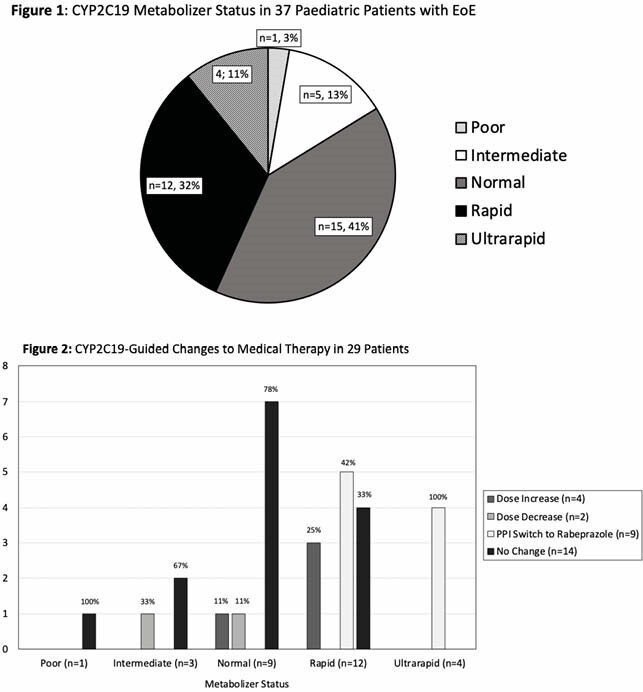

**Funding Agencies:**

Dr. Marcon: J Garfield Campbell Fund, Dr. Hulst: Start-up Funds from the Department of Pediatrics at SickKids

